# Evidence for an A-Modal Number Sense: Numerosity Adaptation Generalizes Across Visual, Auditory, and Tactile Stimuli

**DOI:** 10.3389/fnhum.2021.713565

**Published:** 2021-08-11

**Authors:** Irene Togoli, Roberto Arrighi

**Affiliations:** ^1^International School for Advanced Studies (SISSA), Trieste, Italy; ^2^Department of Neuroscience, Psychology, Pharmacology and Child Health, University of Florence, Florence, Italy

**Keywords:** number sense, numerosity perception, adaptation, tactile perception, cross-modal perception, spatial selectivity

## Abstract

Humans and other species share a perceptual mechanism dedicated to the representation of approximate quantities that allows to rapidly and reliably estimate the numerosity of a set of objects: an Approximate Number System (ANS). Numerosity perception shows a characteristic shared by all primary visual features: it is susceptible to adaptation. As a consequence of prolonged exposure to a large/small quantity (“adaptor”), the apparent numerosity of a subsequent (“test”) stimulus is distorted yielding a robust under- or over-estimation, respectively. Even if numerosity adaptation has been reported across several sensory modalities (vision, audition, and touch), suggesting the idea of a central and a-modal numerosity processing system, evidence for cross-modal effects are limited to vision and audition, two modalities that are known to preferentially encode sensory stimuli in an external coordinate system. Here we test whether numerosity adaptation for visual and auditory stimuli also distorts the perceived numerosity of tactile stimuli (and vice-versa) despite touch being a modality primarily coded in an internal (body-centered) reference frame. We measured numerosity discrimination of stimuli presented sequentially after adaptation to series of either few (around 2 Hz; low adaptation) or numerous (around 8 Hz; high adaptation) impulses for all possible combinations of visual, auditory, or tactile adapting and test stimuli. In all cases, adapting to few impulses yielded a significant overestimation of the test numerosity with the opposite occurring as a consequence of adaptation to numerous stimuli. The overall magnitude of adaptation was robust (around 30%) and rather similar for all sensory modality combinations. Overall, these findings support the idea of a truly generalized and a-modal mechanism for numerosity representation aimed to process numerical information independently from the sensory modality of the incoming signals.

## Introduction

Being able to rapidly estimate the number of objects in the surrounding environment is a fundamental ability for most animal species, humans included. For instance, the ability of selecting the location with more food (e.g., the branch of a tree rich in fruit), or the capacity to make a rapid fight or flight decision (i.e., according to how many predators an animal is facing), have clear implications for survival. Humans, as well as many animal species (Meck and Church, 1983; Emmerton et al., 1997; Kilian et al., 2003; Agrillo et al., 2008, 2011; Rugani et al., 2008) are endowed with a “*sense of number*” that allows them to rapidly—albeit approximately—estimate the number of items in the surrounding space: an Approximate Number System” (ANS). Such mechanism has been reported to be evolutionary ancient (Gallistel, 1990; Dehaene, 1997; Hauser et al., 2000) and innate (Antell and Keating, 1983; Izard et al., 2009) although its acuity has been shown to steadily increase with age in humans (Halberda et al., 2012).

Recent electrophysiological and imaging studies in humans support the existence of a dedicated brain system for the representation of approximate numerical magnitude. For example, studies leveraging on functional magnetic resonance imaging (fMRI) have shown numerosity-related activity in several visual regions throughout the brain dorsal stream, starting from low-level visual areas such as V1-V3 up to high-level associative areas in the parietal cortex (Piazza et al., 2004; Fornaciai and Park, 2018a; Castaldi et al., 2019; DeWind et al., 2019). The processing of numerosity has also been shown to be organized in maps, with a graded tuning to different numerosities resembling the topographic organization of visual sensory inputs in retinotopic maps (Harvey et al., 2013; Harvey and Dumoulin, 2017). The idea of numerosity processing being distributed across several visual areas including early visual cortices has been strengthened by EEG studies showing numerosity-specific brain responses soon after the stimulus onset, to suggest that numerosity is processed (at least partially) also in low-level sensory regions (Park et al., 2016; Fornaciai et al., 2017; Fornaciai and Park, 2018a,b).

Crucially, psychophysical studies have shown that numerosity is subject to adaptation. This is of particular importance, as adaptation is usually considered the hallmark of “primary” perceptual attributes such as, in the visual domain, orientation, color, or size. More specifically, Burr and Ross (2008) showed that after sustained exposure to a dot array containing either a large or small number of dots, the numerosity of the stimulus presented immediately after was strongly distorted, resulting in an under- or over-estimation, respectively (Burr and Ross, 2008). This finding, alongside evidence that numerosity perception obeys Weber’s law (i.e., the threshold varies proportionally with the number of items), led many authors to consider it as a “*primary visual feature*” (see Anobile et al., 2014; Burr et al., 2018).

Additional studies leveraging on adaptation provided important evidence concerning the nature of the brain mechanisms dedicated to numerosity. For instance, it has been reported that numerosity adaptation affects spatial numerosity (i.e., an array of dots simultaneously presented over a region of space) as well as temporal numerosity (i.e., a sequence of flashes presented over a given interval of time) with adaptation to the latter class of stimuli being able to also distort estimates of the numerosity of arrays of dots. Moreover, numerosity adaptation was found to generalize across the visual and auditory modality: adapting to a series of auditory clicks changed the perceived numerosity of sequences of flashes and vice versa, with the adaptation effect being quantitatively similar to that measured within a single sensory modality (vision or audition; Arrighi et al., 2014). This form of cross-modal adaptation has supported the idea of the existence of a generalized, a-modal, mechanism for numerosity processing, possibly located at the top of the numerosity processing stream (i.e., in parietal associative areas like the intraparietal sulcus; Piazza et al., 2004; Harvey et al., 2013), an idea also supported by both neurophysiological studies in the monkey (Nieder, 2012, 2016) as well as imaging studies in humans (Dormal et al., 2010).

Despite the idea of a generalized sense of number, most of the studies on numerosity perception and in particular those dedicated to numerosity adaptation have been limited to the visual or auditory modality. Only recently, a study from our group (Togoli et al., 2021) investigated numerosity adaptation in touch by measuring to what extent numerosity estimates for tactile stimuli are affected by a sustained exposure to slow or rapid sequences of mechanical impulses on the subjects’ finger skin. Adaptation for tactile numerosity turned out in being robust and quantitatively similar to that reported in vision and audition (Togoli et al., 2021). However, so far it has never been investigated whether and to what extent the processing of tactile numerosity affects the processing of numerosity in vision and audition or vice versa. On the one hand, such an interaction should be expected in light of the idea of a truly generalized (or a-modal) number sense meant to process stimulus numerosity regardless of the sensory channels conveying it. On the other hand, it might be that numerosity processing of visual and auditory stimuli converges on a shared mechanism because both systems similarly operate according to an external reference frame exploited to localize and process information of objects in the surrounding environment. Conversely, tactile stimuli are mainly processed *via* a reference frame initially defined in terms of the skin receptors that have been activated by sensory stimulation, which is turned into a spatial reference frame only at a subsequent stage, where sensory information is integrated with body posture—a process termed “tactile remapping.” In other words, in case the interference in numerosity perception across sensory modalities only occurs for sensory channels that leverage on a similar coordinate system, it might be expected that the shared numerosity mechanism between vision and audition would not account for the processing of tactile numerosity information.

To test these hypotheses, we measured the interplay between vision, audition, and touch in numerosity perception by leveraging on the technique of adaptation. We measured the accuracy and precision of numerosity estimates for stimuli presented sequentially (temporal numerosity) in vision, audition, and touch and then measured whether and to what extent these estimates were affected by numerosity adaptation to a relatively high or low quantity of stimuli (i.e., either a low- or a high-frequency stream of stimuli sustained for several seconds) of the same or different sensory modality across several combinations. Namely, we tested: (1) the effect of tactile adaptation on tactile numerical estimates, and a series of cross-modal adaptation conditions concerning; (2) the effect of tactile adaptation on auditory numerosity; (3) the effect of auditory adaptation on tactile numerosity; (4) the effect of tactile adaptation on visual numerosity; and (5) the effect of visual adaptation on tactile numerosity. Furthermore, in one experimental condition (tactile adapters; visual test stimuli) we also tested the role of spatial congruency by measuring adaptation aftereffects when adaptor and test stimuli were superimposed (same spatial position) or with a spatial offset (different spatial positions). If the hypothesis of a truly a-modal number sense is correct, we expect adaptation to be effective irrespective of the modality of adaptor and test stimuli, and to be spatially localized to the adapted location (e.g., see Arrighi et al., 2014; Togoli et al., 2021). Conversely, if cross-modal adaptation could only be observed across similarly “distal” modalities such as vision and audition, then we expect the adaptation to tactile stimuli to affect perceived numerosity of tactile impulses but not that of visual or auditory stimuli. Our results show robust and significant numerosity adaptation effects for all combinations of sensory stimuli, supporting the idea of a truly generalized and a-modal numerosity processing system. Moreover, our results also indicate that cross-modal numerosity adaptation is spatially selective as it vanishes when adaptor and test stimuli are presented in different spatial locations.

## Materials and Methods

### Participants

A total of 16 right-handed subjects participated in the study. The group was composed of six males and 10 females with ages ranging between 23 and 33 years (*M* = 26, SD = 2.67). Six participants were included in each of the five experimental conditions of the present study. Note that the total number of participants does not match the summed sample size considering all the conditions because some of the participants were tested in multiple (but not all) conditions (see below “Behavioral Data Analysis” section). The inclusion criteria for the study required participants to have a normal or corrected-to-normal vision, and the absence of neurological, psychiatric and developmental disorders. The participants were tested separately and signed an informed consent form before participating in the study. All the experimental procedures were approved by the local ethics committee (Comitato Etico Pediatrico Regionale—Azienda Ospedaliero-Universitaria Meyer—Firenze FI) and were in line with the Declaration of Helsinki. Note that the sample size of the present study was decided *a priori* based on the cross-modal adaptation effects measured in Arrighi et al. (2014). Namely, we took the average effect size yielded by 2-Hz and 8-Hz adaptation to visual stimuli on numerical estimates of the sequence of sounds as well as the effect of auditory adaptation on visual numerical estimates. Considering this average effect size (Cohen’s *d* = 2.92), a power of 99%, and a two-tailed distribution, the estimated minimum sample size was five subjects.

### Apparatus and Stimuli

**Figure 1:**
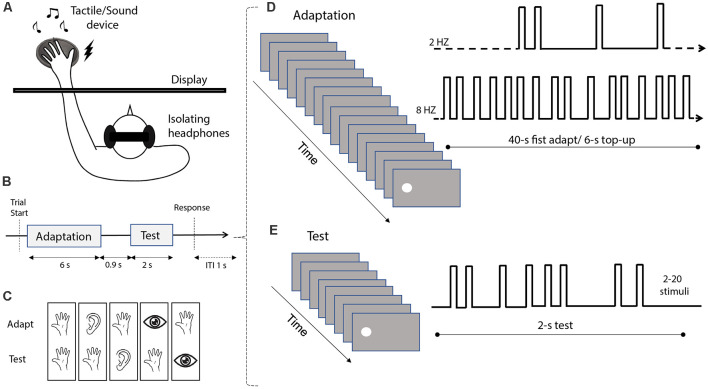
Stimuli and experimental procedure. **(A)** Schematic depiction of the experimental setup. Participants seated in front of a monitor screen, with their hands positioned on the audio-tactile device located behind the screen. To avoid receiving auditory feedback during tactile stimulation, the subjects wore isolating headphones. **(B)** Depiction of the trial sequence. Each trial included an adaptation phase (6 s; with the exception of the first trial where the adaptation duration was 40 s) followed by a test phase (2 s), with an inter-stimulus interval of 0.9 s. At the end of each trial, participants were asked to estimate the numerosity of the test stimulus. After providing a response, the next trial started after an inter-trial interval (ITI) of 1 s. **(C)** The different combinations of adaptation and test stimulus modality (from left to right: tactile-tactile, audio-tactile, tactile-audio, visual-tactile, and tactile-visual). **(D)** Example of the adaptation sequence. Two different types of adaptation were tested in each condition: “low” adaptation, involving stimulation at 2 Hz, and “high” adaptation, involving stimulation at 8 Hz. In the first trial, adaptation was delivered for 40 s, while in each following trial we delivered a “top-up” adaptation of 6 s. **(E)** Example of the test stimulus sequence. Each test sequence included 2–20 individual stimuli, delivered within an interval of 2 s. Note that these examples depict the presentation of visual adaptor or test stimuli, but in different conditions, the sequences could involve also sounds or vibrotactile pulses. The examples are not depicted in scale.

The experimental setup included a 17-inches touch screen monitor (resolution 1,280 × 1,024 pixels; refresh rate 60 Hz; LG-FLATRON L1732P), used to present the visual stimuli, and a Clark Synthesis Tactile Sound Transducer (TST429 platinum), positioned behind the screen (in a position corresponding to the location of the visual stimuli on the screen in all experimental conditions except that in which tactile and visual stimuli were presented spatially separated), used to deliver both auditory and tactile stimuli (Figure 1A). The tactile sound transducer was composed of a speaker with a rubber ball mounted on top of it, used to convey the speaker vibrations to the hand of the participant. Additionally, the transducer was mounted on an inflatable cushion to avoid the additional noise of vibrations spreading to the table.

All the stimuli used in the different conditions of the present study were generated using Matlab (version R2010a) and the Psychophysics Toolbox (Brainard, 1997; Pelli, 1997), on a computer running Windows 7. The visual stimuli were white discs of 5° diameter, displayed 8° to the left or to the right of the central fixation point (see below “Procedure” section). The auditory and tactile stimuli were both presented through the tactile sound transducer device positioned behind the screen, centered at 8° from the center of the screen. Auditory stimuli were 500-Hz sine waves, with a 5-ms ramp at the onset and offset played at an intensity of around 75 dB. Tactile stimuli were generated through 50-Hz sine waves, a frequency specifically chosen to elicit vibrations to the subjects’ skin without being audible through the insulating headphones wore by participants (see below).

In all conditions, the test stimuli were pseudo-random sequences of flashes (i.e., white discs), tones, or vibrotactile pulses (Figure 1E), with numerosity ranging from 2 to 20 stimuli. However, during data analysis, we only considered numerosities from 5 to 15 to avoid edge effects at the highest extreme, and the subitizing range (numerosity <5) in the lowest extreme as estimates in the subitizing range are known to be errorless and not susceptible to adaptation (Anobile et al., 2020). Each stimulus in the sequence was presented for 40 ms. To minimize the temporal regularity of the sequence, the ISI between any two consecutive stimuli in each sequence was randomly determined, with the constrain of a minimum ISI of 40 ms between two consecutive stimuli, and an overall sequence duration of 2 s. Adaptor stimuli were similarly pseudo-random sequences of flashes, tones, or vibrotactile pulses (Figure 1D). Each stimulus in the sequence lasted for 40 ms. Two different adaptation conditions were defined. In the *low* adaptation condition, adaptor sequences had a frequency of 2 stimuli/s (2 Hz), while in the *high* adaptation condition the adaptor had a frequency of 8 stimuli/s (8 Hz). These adaptation frequencies were chosen to be consistent with previous studies from our group showing robust adaptation effects (Arrighi et al., 2014; Togoli et al., 2021). Note that following previous studies (e.g., Arrighi et al., 2014), in our experimental design we induced adaptation effects *via* a prolonged presentation of a sequence of stimuli presented either with a low (2 Hz) or high (8 Hz) frequency. This technique has been already shown to be highly effective in previous studies concerning perceived numerosity (Arrighi et al., 2014; Anobile et al., 2016; Togoli et al., 2020, 2021), and was also adopted to avoid potential positive (i.e., opposite to adaptation) “serial dependence” effects reported to occur with a shorter stimulus presentation (see for instance Fornaciai and Park, 2019a).

### Procedure

The experiment was performed in a quiet and dimly lit room, with participants wearing insulating headphones throughout the session, which allowed the auditory stimuli to be perceived but prevented the auditory feedback from the tactile stimuli. In all conditions, participants performed a numerosity estimation task of visual, auditory, or tactile impulses in a sequence, after being adapted to either visual, auditory, or tactile stimuli. More specifically, while participants fixated on a central fixation point, the adaptor stimulus was delivered first, followed by the test stimulus after an ISI of 900 ms (Figure 1B). In the first trial of each block, the adaptor stimulus was presented for 40 s. In the following trials, we delivered a shorter top-up adaptor stimulus for 6 s. Participants were instructed that the first sequence in each trial was not relevant for the task, while they had to attend to and report the numerosity of the stimuli in the second sequence. At the end of the trial, a virtual number pad appeared on the screen, and participants were instructed to dial the number of stimuli in the sequence they had perceived by using the computer mouse. The response number was displayed on the screen, and participants pressed another button to confirm their response, then the next trial started after 1 s.

Participants performed a total of five conditions (Figure 1C) involving a different combination of adaptation and test sensory modalities (tested separately). The conditions were as follows. (1) A purely tactile condition (“Tact-Tact”), in which both adaptor and test stimuli were sequences of tactile impulses. (2) A tactile-auditory condition (“Tact-Aud”), in which the adaptor was tactile, and the test stimulus was a sequence of sounds. (3) An auditory-tactile condition (“Aud-Tact”) in which the adaptor was auditory, and the test stimulus was tactile. (4) A visual-tactile condition (“Vis-Tact”), entailing visual adaptation and tactile test stimuli. (5) A tactile-visual condition (“Tact-Vis”) with tactile adaptation and visual test stimuli. This last condition was further divided into two different sub-conditions (interleaved within the same blocks), with test stimuli being either spatially matched (Matched position), or presented with a 16° spatial offset (Unmatched position). The two sub-conditions were devised to test for the spatial selectivity of the adaptation effect across the tactile and visual modality.

In the Tact-Tact, Tact-Aud, Aud-Tact, and Vis-Tact conditions, participants performed 7–9 blocks of 20 trials. In the Tact-Vis condition, instead, participants performed five blocks of 40 trials (with the exception of one participant who performed four blocks of 40 trials due to equipment failure). To avoid the different adaptation conditions to interfere with each other, they were performed in different days, with their order randomized across participants. Before the start of each condition, participants were familiarized with the stimuli by performing a few trials without adaptation. No feedback was provided concerning the participants’ responses in any of the conditions. Each session took about 120 min, and participants were allowed to take frequent breaks between different blocks.

### Data Analysis

As a measure of accuracy in the numerosity estimation task, we computed for each subject, in each experimental condition, the average numerical estimate for each level of numerosity (5–15). Precision was instead measured in terms of Weber’s fraction, defined as the standard deviation of numerical estimates divided by the average estimate (WF = σ_est_/μ_est_), again computed separately for each subject and condition. To assess the effect of different types of adaptation on numerical estimates, we first performed a series of two-way repeated measures ANOVAs within each condition, with factors “numerosity” (5–15), and “adaptation” (low adaptation vs. high adaptation). Interactions between different factors observed in the ANOVAs were followed up with paired t-tests between low and high adaptation, at each level of the numerosity range. Note that to the purpose of this series of tests, in the Tact-Vis condition we only considered the case in which visual and tactile stimuli were presented in the same spatial position. A comparison between the matched and unmatched sub-conditions was performed separately to assess the spatial selectivity of the effect (see below).

We also assessed subjects’ precision in the estimation task in terms of WFs for all conditions (defined by the sensory modality of adapters and test stimuli) and across the two kinds of adaptation (i.e., low vs. high). Statistical tests on precision were carried out with a two-way (independent-samples) ANOVA on WFs averaged across numerosities, with factors “condition” (Tact-Tact, Tact-Aud, Aud-Tact, Tact-Vis, Vis-Tact), and “adaptation” (low vs. high).

Moreover, to better assess the magnitude of effects across different conditions, and compare them directly, we computed an adaptation effect index (AI) as follows:

$$AI=\left(\frac{{\bar{\text{PN}}}_{\text{low}}-{\bar{\text{PN}}}_{\text{high}}}{{\bar{\text{PN}}}_{\text{high}}}\right)*100$$

where ${\bar{\text{PN}}}_{\text{low}}$ represents the average numerical estimate across all numerosities after low adaptation, and ${\bar{\text{PN}}}_{\text{high}}$ the average numerical estimate after high adaptation. To compare the effect across different conditions, first, we performed a one-way independent samples ANOVA on the AIs, and then we compared individually each condition with a series of independent samples t-tests. To account for multiple comparisons, we applied a false-discovery rate (FDR) procedure with *q* = 0.05.

Finally, in the TactVis condition, we assessed the spatial selectivity of the effect by comparing the adaptation effects when adaptor and test stimuli were superimposed or spatially separated. First, we performed a three-way repeated-measures ANOVA with factors “numerosity” (5–15), “adaptation” (low vs. high), and “test location” (matched vs. unmatched). This test was followed up by *post hoc* tests to address interaction effects. Finally, we directly compared the magnitude of the effect measured in the two conditions in terms of the adaptation index. To do so, we performed two one-sample t-tests against the null hypothesis of zero effect, and a paired t-test comparing the effect in the matched and unmatched conditions.

Note that similarly to previous studies from our group (Arrighi et al., 2014; Anobile et al., 2016, 2020; Togoli et al., 2020, 2021), the adaptation effect here is computed considering two opposite adaptation conditions, rather than considering the difference from a baseline condition without adaptation. Although performing a baseline condition might provide more evidence concerning the adaptation-induced distortion of perceived numerosity compared to the absence of adaptation, it could introduce biases in the estimation of the effect. Indeed, having different sequences of stimuli (i.e., with or without the presentation of the adaptor) might provide different biases through time-order errors (i.e., the systematic under- or over-estimation of the first stimulus in a sequence; see for instance Hellström, 1985). For this reason, we chose not to add a baseline condition, and compute the adaptation effect as the difference between two opposite adaptation conditions.

## Results

**Figure 2:**
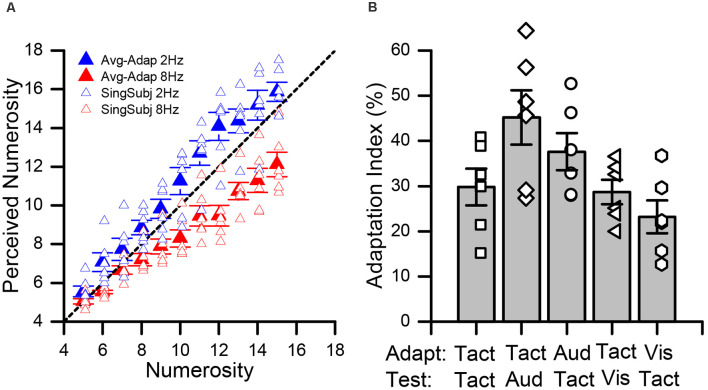
Effect of adaptation in the tactile and cross-modal conditions. **(A)** Average numerical estimates for each numerosity (from 5 to 15), in the pure tactile (Tact-Tact) condition. Data relative to the low adaptation condition (2 Hz) are shown in blue whilst those for high adaptation (8 Hz) in red. Individual data (averaged over trials) are shown by the empty symbols whilst bold filled symbols indicate averages across participants. **(B)** Average adaptation effect indexes (AIs) across the different conditions. The empty data points show the adaptation effect for all participants. Error bars represent SEM.

In all experimental conditions, we measured subjects’ average estimates for each numerosity for both high and low adaptation. Figure 2 shows data for the pure tactile experiment (Tact-Tact), in which both the adapter and test stimuli were tactile. As a consequence of adaptation to sequences of few tactile impulses (2 Hz), all subjects showed a tendency to overestimate the numerosity of the presented test stimuli (blue data point in Figure 2A). On the contrary, after adaptation to sequences entailing numerous stimuli (8 Hz), subjects showed a robust tendency to underestimate the numerosity of the test stimuli (red data points in Figure 2A). This pattern of results did hold for all possible combinations of stimulus sensory modalities (Supplementary Figure 1) and it is in line with the effects of numerosity adaptation reported in previous studies (e.g., Burr and Ross, 2008; Arrighi et al., 2014; Togoli et al., 2021).

To assess the significance of the adaptation effect across the different conditions, we performed a series of two-way repeated measures ANOVAs on the average numerical estimates, with factors “numerosity” (5–15), and “adaptation” (low vs. high). In all the conditions, as expected, we observed a significant main effect of numerosity (TactTact: *F*_(10, 55)_ = 157.9, *p* < 0.001, $\eta_{\text{p}}^{2}$ = 0.97; TactAud: *F*_(10, 55)_ = 127.8, *p* < 0.001, $\eta_{\text{p}}^{2}$ = 0.96; AudTact: *F*_(10, 55)_ = 169.2, *p* < 0.001, $\eta_{\text{p}}^{2}$ = 0.97; TactVis: *F*_(10, 55)_ = 109.7, *p* < 0.001, $\eta_{\text{p}}^{2}$ = 0.96 and VisTact: *F*_(10, 55)_ = 134.5, *p* < 0.001, $\eta_{\text{p}}^{2}$ = 0.96). Moreover, we also observed a significant main effect of adaptation, again across all the conditions (TactTact: *F*_(1, 5)_ = 52.5, *p* < 0.001, $\eta_{\text{p}}^{2}$ = 0.91; TactAud: *F*_(1, 5)_ = 79.3, *p* < 0.001, $\eta_{\text{p}}^{2}$ = 0.94; AudTact: *F*_(1, 5)_ = 89.4, *p* < 0.001, $\eta_{\text{p}}^{2}$ = 0.95; TactVis: *F*_(1, 5)_ = 136.9, *p* < 0.001, $\eta_{\text{p}}^{2}$ = 0.96 and VisTact: *F*_(1, 5)_ = 38.1, *p* = 0.001, $\eta_{\text{p}}^{2}$ = 0.88). Furthermore, we also observed in all conditions a significant interaction between the two factors (TactTact: *F*_(10, 55)_ = 7.6, *p* < 0.001, $\eta_{\text{p}}^{2}$ = 0.60; TactAud: *F*_(10, 55)_ = 12.2, *p* < 0.001, $\eta_{\text{p}}^{2}$ = 0.71; AudTact: *F*_(10, 55)_ = 15.7, *p* < 0.001, $\eta_{\text{p}}^{2}$ = 0.76; TactVis: *F*_(10, 55)_ = 6.7, *p* < 0.001, $\eta_{\text{p}}^{2}$ = 0.57 and VisTact: *F*_(10,55)_ = 5.2, *p* < 0.001, $\eta_{\text{p}}^{2}$ = 0.51), to suggest differences in the strength of adaptation for different levels of numerosity. Indeed, looking at Figure 2A (Supplementary Figure 1), it is evident that adaptation is more effective at relatively high numerosities. However, a series of *post hoc* paired *t*-tests (corrected for multiple comparisons with a false discovery rate, FDR, procedure, with *q* = 0.05) within each numerosity showed a statistically significant difference between numerical estimates after low vs. high adaptation for the majority of the tested numerosities with just few exceptions. In the Tact-Tact and Tact-Aud condition, all comparisons were statistically significant (max FDR-adjusted *p*-value = 0.044). In the Aud-Tact and Tact-Vis condition, all comparisons were significant (max FDR-adjusted *p*-value = 0.049 and 0.043, respectively), with the exception of the numerosity level 5 in the Aud-Tact condition (adj-*p* = 0.21) and the numerosity level 6 in the Tact-Vis condition (adj-*p* = 0.06). Finally, in the Vis-Tact condition, all the comparisons were significant (max adj-*p* = 0.016), with the exception of numerosity level 5 and 7 (adj-*p* = 0.11 and 0.12, respectively).

In addition to subjects’ accuracy in numerosity estimates (i.e., the mean numerical estimates, reflecting perceived numerosity), we also measured their precision in terms of Weber’s fraction (WF; see “Materials and Methods” section). We measured whether there was any difference in precision across the different conditions and as a function of the adaptation frequency (i.e., low vs. high). The average WFs across the different conditions are shown in Supplementary Figure 2. To this aim, we performed a two-way (independent samples) ANOVA on WF measures averaged across numerosities, with factor “condition” (Tact-Tact, Tact-Aud, Aud-Tact, Tact-Vis, Vis-Tact), and “adaptation” (low vs. high). The results showed neither a main effect of condition (*F*_(4,25)_ = 1.01, *p* = 0.41), nor a main effect of adaptation (*F*_(1,5)_ = 0.006, *p* = 0.94), and no interaction between the two factors (*F*_(4,25)_ = 0.17, *p* = 0.95). Given that WFs reflects variability in subjects’ responses and this is meant to reflect the noise related to the perceptual process, we can conclude that in none of the conditions the two kinds of adaptation differed in providing a different amount of variability in numerosity processing.

Moreover, in order to obtain a direct comparison of the magnitude of the adaptation effect and compare the effects observed in different conditions, we calculated an adaptation effect index (AI) as the normalized difference between numerical estimates after low and high adaptation, turned into percentage (see formula 1 in the “Data Analysis” section). The average AIs across the different conditions tested are shown in Figure 2B. Overall, we observed robust adaptation effects across all conditions. Indeed, a series of one-sample *t*-tests (against the null hypothesis of zero effect; corrected with FDR) showed that the effect is significant in all tested conditions (Tact-Tact: *t*_(5)_ = 7.32, adjusted-*p* < 0.001, Cohen’s *d* = 2.99; Aud-Tact: *t*_(5)_ = 9.15, *p* < 0.001, *d* = 3.07; Tact-Aud: *t*_(5)_ = 7.52, *p* < 0.001, *d* = 3.72; Tact-Vis: *t*_(5)_ = 10.64, *p* < 0.001, *d* = 4.34; Vis-Tact: *t*_(5)_ = 6.37, *p* = 0.001, *d* = 2.60). Then, we performed a one-way independent samples ANOVA (with factor “condition”) to compare the magnitude of adaptation across all the combinations of sensory modalities of adapting and test stimuli. The results show a significant main effect of condition (*F*_(4, 25)_ = 4.1, *p* = 0.01, $\eta_{\text{p}}^{2}$ = 0.40), suggesting that the adaptation magnitude might actually vary across conditions depending on which modality adaptor and test stimuli belonged to. To further investigate this, we ran a series of pairwise independent-sample *t-tests* comparing the conditions against each other. Again, to account for multiple comparisons, we applied an FDR procedure with *q* = 0.05. The results showed no statistically significant differences across conditions after correcting for multiple comparisons (max *t*-value = 3.14, min adjusted *p*-value = 0.10), suggesting that numerosity adaptation effects across vision, audition, and touch are quite similar in magnitude, regardless of the sensory modality of the adapting and test stimuli.

While the effect of numerosity adaptation within different modalities (visual, auditory, tactile) has been shown to be spatially localized (Arrighi et al., 2014; Togoli et al., 2021), is the cross-modal effect similarly selective for the position of the stimuli? The hypothesis of a truly a-modal numerosity processing system predicts indeed that adaptation should show similar properties—included spatial selectivity—irrespective of the sensory modality of the adaptor and test stimuli, and irrespective of whether the two stimuli belong to the same or different modalities. To address this prediction, we divided the Tact-Vis condition into two sub-conditions. In one condition the visual test stimulus was presented spatially superimposed with the position of the tactile adapter (matched condition), whilst in the other, it was presented with a horizontal spatial offset (unmatched condition). The prediction was straightforward: if the effect is spatially selective, we would expect a significant adaptation effect only when adaptor and test stimuli are presented in a spatially matched position.

**Figure 3:**
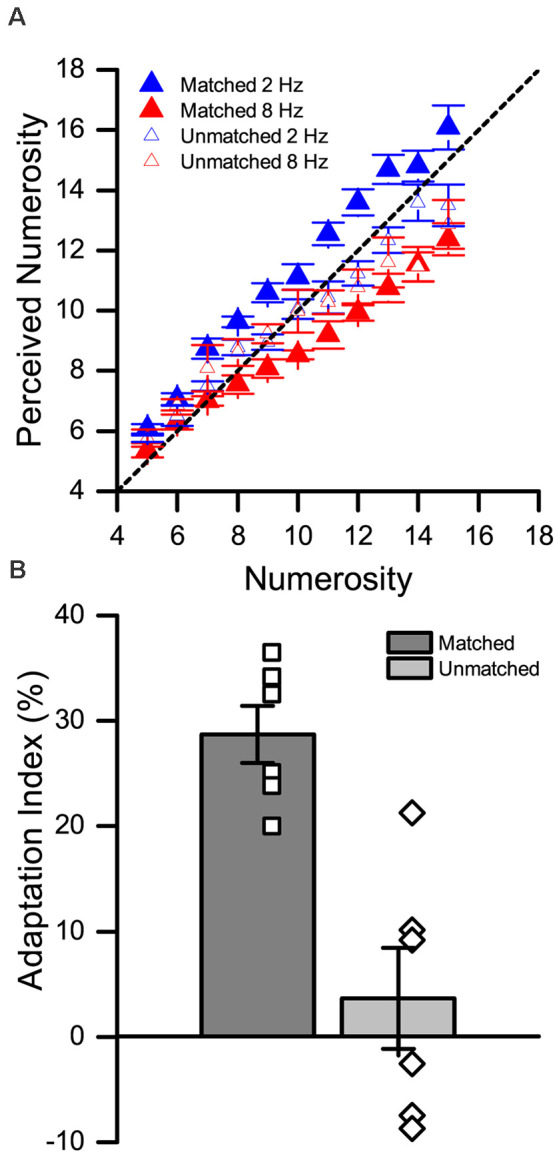
Spatial selectivity in the cross-modal tactile-visual (Tact-Vis) condition. **(A)** Average numerical estimates for each numerosity presented in the low (blue triangles) and high (red triangles) matched condition. The empty symbols correspond to the unmatched condition and the filled symbols to the matched condition. **(B)** Average adaptation effect indexes (AIs) in the matched (dark gray bar) and unmatched (light gray bar) condition. The empty symbols show the individual estimates of the effect. Error bars represent SEM.

The results are shown in Figure 3. To assess the effect of adaptation in the matched and unmatched condition, we first performed a three-way repeated measures ANOVA, with factors “numerosity” (5–15), “adaptation” (low vs. high), and “test position” (matched vs. unmatched). The results showed a main effect of numerosity (*F*_(10,55)_ = 139.6, *p* < 0.01, $\eta_{\text{p}}^{2}$ = 0.97), a main effect of adaptation (*F*_(1,5)_ = 26.9, *p* = 0.003, $\eta_{\text{p}}^{2}$ = 0.84), and a main effect of test position (*F*_(1,5)_ = 6.6, *p* = 0.004, $\eta_{\text{p}}^{2}$ = 0.57). A significant two-way interaction was observed between numerosity and adaptation (*F*_(10,55)_ = 5.03, *p* < 0.01, $\eta_{\text{p}}^{2}$ = 0.50), and between adaptation and position (*F*_(1,5)_ = 27.16, *p* < 0.01, $\eta_{\text{p}}^{2}$ = 0.84). On the contrary, no significant interaction was found between numerosity and position (*F*_(10,55)_ = 1.57, *p* = 0.14). Finally, we observed a significant three-way interaction between numerosity, adaptation and position (*F*_(10,55)_ = 2.21, *p* = 0.03, $\eta_{\text{p}}^{2}$ = 0.31).

To address the nature of this three-way interaction, we performed a series of (FDR-corrected) *post hoc* paired *t*-tests comparing numerical estimates after low vs. high adaptation at each numerosity level, separately for the matched and unmatched condition. In the matched condition we observed a statistically significant effect of adaptation (i.e., a significant difference between numerical estimates after low vs. high adaptation) for each numerosity (max adj-*p* = 0.043) except one (6, adj-*p* = 0.062). Conversely, in the unmatched condition, we did not observe any significant difference in numerical estimates induced by adaptation at any numerosity (min adj-*p* = 0.84).

Finally, we also computed the AI for the matched vs unmatched condition, and compared them. As shown in Figure 3B, while in the matched condition the adaptation effect is robust, it is almost null when adapting and test stimuli were presented in different spatial locations. A two one-sample t-tests (against zero) showed that while the effect in the matched condition was significantly higher than zero (*t*_(5)_ = 10.64, *p* < 0.001, *d* = 4.34), the effect was not significant in the unmatched condition (*t*_(5)_ = 0.76, *p* = 0.48, *d* = 0.31). In line with that, a paired t-test further showed that the effect in the matched condition is significantly higher compared to the unmatched condition (*t*_(5)_ = 5.32, *p* = 0.003, *d* = 2.17).

## Discussion

In the present study, we tested the idea of a generalized and a-modal mechanism to process numerosity in the human brain by measuring the effect of adaptation across different sensory modalities. Participants were asked to estimate the numerosity of either a sequence of brief flashes, tones, or vibrotactile pulses. Crucially, before the presentation of these test stimuli, participants were adapted to sequences of either flashes, tones, or vibrotactile pulses, at different frequencies entailing a relatively low or relatively high number of events (i.e., low and high adaptation condition, respectively). The conditions tested included a purely tactile condition (tactile adaptation on tactile numerical estimates; Tact-Tact), and a series of cross-modal combinations: tactile adaptation on auditory or visual numerical estimates (Tact-Aud and Tact-Vis, respectively) and auditory or visual adaptation on tactile numerical estimates (Aud-Tact and Vis-Tact, respectively). Overall, our results show robust and significant adaptation effects: a period of 2 Hz stimulation yielded robust overestimation of perceived numerosity of the subsequent test stimulus, while 8 Hz adaptation caused a relative underestimation. Importantly, we show that adaptation aftereffects were quantitatively similar across all the combinations of stimulus sensory modalities.

Despite decades of studies, the brain mechanisms supporting the ability to rapidly and approximately estimate quantities of items—an ability fundamental for survival—remain unclear. In recent years, neuroimaging studies have started to uncover the brain areas and the processing stages linked to numerosity perception. For instance, fMRI studies on visual numerosity perception have shown a pathway for the processing of approximate numerical information starting from the early stations of the visual cortex, towards high-level associative cortices in the parietal cortex. Indeed, although the parietal cortex is the most consistently reported brain region associated with numerosity perception (e.g., Piazza et al., 2004; Dormal and Pesenti, 2009; Harvey et al., 2013; Borghesani et al., 2019; but see Cavdaroglu et al., 2015; and Cavdaroglu and Knops, 2019)—and thus it is considered the core of its processing pathway—other studies have started to uncover the contributions of earlier sensory areas. Indeed, early visual areas such as V1, V2, and V3, have started to be increasingly reported in fMRI as associated with numerosity processing (Fornaciai and Park, 2018a; Castaldi et al., 2019; DeWind et al., 2019). Electroencephalography (EEG) studies further support this idea of a numerosity processing pathway starting from early sensory areas, at least in vision. Namely, it has been recently shown that numerosity-related evoked activity emerges as early as 75–100 ms after the onset of a stimulus (i.e., C1 component), and from areas like V1-V3 (Park et al., 2016; Fornaciai and Park, 2017, 2018a; Fornaciai et al., 2017; Van Rinsveld et al., 2020), and continues through later latencies (i.e., 180–200 ms, P2p component; e.g., Temple and Posner, 1998; Libertus et al., 2007; Hyde and Wood, 2011). All these neuroimaging studies thus provide evidence for the existence of a dedicated brain network for the processing of approximate numerical information.

Studies at the behavioral level further support the existence of brain mechanisms specific to numerosity. For instance, it has been shown that visual perception is more sensitive to numerosity than to other non-numerical visual attributes like texture-density (Anobile et al., 2016; Cicchini et al., 2016), suggesting indeed the existence of dedicated brain mechanisms for numerosity processing (although non-numerical attributes may still contribute to numerosity perception, see for instance Dakin et al., 2011 and Leibovich et al., 2017, for alternative accounts). Furthermore, numerosity perception has also been shown to be modulated by the spatio-temporal properties of the stimuli and by motion (Fornaciai and Park, 2018c; Fornaciai et al., 2018), suggesting again a role for relatively early sensory areas. Most notably, it has been shown that numerosity perception is subject to perceptual adaptation (Burr and Ross, 2008; see Kohn, 2007 for a review on adaptation). Perceptual adaptation is indeed considered the hallmark of a fundamental—*primary*—perceptual attribute (i.e., like for instance orientation, color, contrast, or motion; Burr and Ross, 2008; Grasso et al., 2021; but see Durgin, 2008, for an alternative account). Interestingly, numerosity adaptation has been shown to not be limited to vision, but to also extend to other modalities, like audition (Arrighi et al., 2014; Togoli et al., 2020) and touch (Togoli et al., 2021). Even more striking, is the observation of cross-modal adaptation: adapting to a stream of auditory events can affect the perceived numerosity of visual stimuli, and vice versa (Arrighi et al., 2014; see also Anobile et al., 2016, 2020, and Togoli et al., 2020; Maldonado Moscoso et al., 2020, for adaptation effects across the motor and sensory domain).

The observation of cross-modal adaptation has suggested the existence of a generalized, a-modal, number sense (Arrighi et al., 2014), most likely implemented at the top of the numerosity processing pathway (i.e., parietal cortex; see for instance Castaldi et al., 2016). These results nicely complement the neurophysiological results in the monkey brain and imaging data in the human brain. For example, neurons in the ventral intraparietal sulcus (IPS) of monkeys have been reported to encode numerosity for both auditory and visual sensory modalities to suggest that numerosity information eventually converges to a more abstract representation (Nieder, 2012, 2016). Similarly, in humans, a right lateralized frontoparietal circuit activated by both auditory and visual number sequences, has been reported (Piazza et al., 2006).

The present results further extend and support the idea of a generalized number sense, by showing that adaptation occurs across a wide range of cross-modal conditions. Previous results have been indeed limited to the auditory and visual modalities—two modalities that are both characterized by the need of processing distal stimuli (i.e., stimuli originating away from the sensory organ transducing their energy). Such similarity between these two modalities raised the question of whether the number sense is truly amodal, as the cross-modal adaptation may remain limited to auditory and visual stimulation. A truly amodal processing system would instead predict similar adaptation effects irrespective of the sensory modality through which adaptor and test stimuli are delivered—even when a quite different modality, like touch, is involved. And this is exactly what our results show: adaptation generalizes across several different cross-modal combinations, and works similarly irrespective of the sensory modality involved. In terms of the brain processing stage probed by adaptation, our results suggest that adaptation occurs at a level in the processing hierarchy at which signals from different sensory modalities interact with each other. In previous studies, cross-sensory interactions have been observed at multiple levels of “uni-sensory” pathways, and as early as the primary sensory cortices of different modalities (e.g., Laurienti et al., 2002; Schroeder and Foxe, 2005; Mishra et al., 2007; Sperdin, 2009; Vasconcelos et al., 2011). However, results from both the present and previous studies congruently suggest that numerosity adaptation mainly occurs in higher-order integrative cortical areas such as the parietal cortex (Castaldi et al., 2016). In line with that, previous results from our group show that numerosity adaptation also generalizes across the perceptual and motor system: adaptation to a series of self-generated tapping movements distorts the perceived numerosity of subsequently-presented visual (Anobile et al., 2016) or auditory (Togoli et al., 2020) stimuli. Thus, numerosity adaptation seems to occur at the converging point of modality-specific sensory pathways and motor signals, making the parietal cortex the best candidate locus for the brain mechanisms involved in numerosity adaptation (e.g., Iacoboni, 2006; Tosoni et al., 2008).

The fact that we did not observe a significant difference across the different adaptation conditions is in line with the idea of a high-level mechanism mediating the number sense. This result is particularly interesting, as one may intuitively expect to observe the stronger and more robust effect in the uni-modal condition (i.e., involving only tactile stimulation). The observation of no significant differences across the different conditions thus supports the idea of a truly generalized, a-modal number sense, whereby the processing of different numerical quantities and adaptation effects are independent of the sensory modality the numerosity information originally belonged to. However, caution is in order when interpreting the non-significance of this result. Indeed, our study was designed to detect a significant adaptation effect against the null hypothesis of zero effect, and not a subtler difference in the level of effect across different conditions, since we did not have a clear a-priori hypothesis concerning this point. Our design may thus lack the necessary power to detect a significant difference across conditions, leaving this point as an open question that should be addressed by future studies.

Furthermore, we also show that tactile adaptation has a spatially-localized effect on visual stimuli, similar to previous studies showing spatially localized adaptation effects in vision and in the tactile modality (Arrighi et al., 2014; Togoli et al., 2020; see also Anobile et al., 2020). In other words, in the Tact-Vis condition, we show that tactile adaptation can affect the perceived numerosity of a visual stimulus only when such a stimulus is presented in the same position as the adaptation. This is particularly important, for two reasons. First, it shows that numerosity processing involves the same spatio-temporal computations in different modalities, and suggests a common encoding of numerical information from the two modalities within a similar topographic representation of external space. Second, it suggests that the effect is perceptual in nature, and not a cognitive or decisional effect, as in this latter case the effect of numerosity adaptation would be expected to occur regardless of the position of the stimuli, with no spatial selectivity (Arrighi et al., 2014).

It is important to note that the generalization across different sensory modalities seems to be a specific property of numerosity adaptation. Indeed, it has been shown that a different effect inducing an attractive bias based on the recent history of stimulation (i.e., serial dependence; Fischer and Whitney, 2014; Fornaciai and Park, 2018c) does not show such generalization. Namely, while serial dependence in numerosity perception entails a spatially-localized effect (Fornaciai and Park, 2018c) and works across sequentially and simultaneously presented visual stimuli (Fornaciai and Park, 2019a), similarly to adaptation, it does not extend between auditory and visual stimuli (Fornaciai and Park, 2019a). However, adaptation and serial dependence likely entail widely different neurophysiological and functional mechanisms (see for instance Fornaciai and Park, 2019b), which may explain this difference. Addressing and comparing these different mechanisms thus represents an interesting open question for future studies.

Finally, another important point to consider is whether the temporal frequency (or rate) *per se* of the stimuli—rather than their numerosity—might have played a role in the observed results. Indeed, our adaptation sequences were defined by different temporal frequencies: 2 Hz (low) vs. 8 Hz (high). However, although numerosity and temporal frequency are potentially confounded in this adaptation design, it is unlikely that temporal frequency adaptation could explain the observed results, for three main reasons. First, we need to consider the relation between the frequency of adaptor and test stimuli. Indeed, while the adaptor stimuli had either a frequency of 2 or 8 Hz, the frequency of the test stimuli (considering that they were presented in a 2-s interval) varied with numerosity, spanning from 2.5 Hz to 7.5 Hz (respectively for 5 and 15 stimuli). If the effect was mediated by temporal frequency, we would thus expect a variable pattern of adaptation effects at different numerosities: the effect should have increased with the difference in frequency between adaptor and test stimuli. Namely, 2 Hz adaptation should be minimally effective on low-numerosity stimuli, while it should have the strongest effect at higher numerosities. The opposite is true for 8 Hz adaptation, which should have the maximum effect at low numerosities and the minimum effect at higher numerosity. However, no such pattern is evident neither in our results (see Figures 2A, 3A and Supplementary Figure 1), nor in previous reports leveraging on the same paradigm (Arrighi et al., 2014; Togoli et al., 2021). Second, previous results show no transfer of frequency adaptation across different modalities (Motala et al., 2018), or cross-modal effects that are tightly tuned to the frequency band of the stimuli (i.e., a 4 Hz stimulus is strongly affected by a 5 Hz adaptor, but less so by adaptors of slightly different frequency). Third, temporal frequency adaptation is usually considered a very low-level effect, occurring at the earliest levels of sensory processing like the lateral geniculate nucleus (LGN) in vision (Tan and Yao, 2009), and the primary somatosensory cortex (S1) in touch (Romo and Salinas, 2003). Such early locus of temporal frequency adaptation is thus at odds with the cross-modal transfer observed in the present study. For all these reasons, we believe that the observed results are more in line with a numerosity adaptation effect, rather than temporal frequency adaptation. Nevertheless, another aspect worth it mentioning is that in this specific adaptation protocol the effect does not seem to be modulated by the relative numerosity of the adaptor and test sequences. For instance, one may expect the effect to be modulated by the ratio between adaptor and test (e.g., Piazza et al., 2004). Our results instead show a consistent pattern of adaptation effects in the low and high adaptation conditions, with the magnitude of adaptation roughly increasing with increasing test numerosity. This shows that—in line with previous studies employing a similar methodology (Arrighi et al., 2014; Togoli et al., 2020)—the effect is indeed not modulated by the ratio of the stimuli. If so, we should have instead observed a quite different pattern of effects (i.e., the effect of high adaptation should have peaked at lower test numerosities, and vice versa for the low adaptation). A possibility explaining this feature of the effect might be the relatively long duration of the adaptor stimuli, preventing the visual system from tracking the total numerosity of the adaptor stimuli throughout their presentation interval. However, since we kept the duration of the adaptation sequences constant, our results could not clarify this point, which thus remains another interesting open question for future studies.

Finally, besides the specific mechanisms of numerosity perception, our results are consistent with a broader view of perception as being largely multisensory (e.g., Pascual-Leone and Hamilton, 2001). Stimulation from the external environment is indeed intrinsically multisensory, and object representation has been observed to be systematically facilitated in the presence of multisensory information (e.g., Amedi et al., 2005). Multisensory integration has been shown to affect even the very low-level properties of a stimulus, like for instance the position of a visual flash of light strongly biasing the perceived position of a sound, with multisensory information being integrated in a statistically optimal fashion (i.e., Alais and Burr, 2004). In line with this idea, we show that numerosity—which could be considered a primary perceptual feature (Anobile et al., 2016)—is processed in an intrinsically multi-modal fashion, with the effect of adaptation (e.g., see Kohn, 2007 for a review) occurring independently from the sensory modality of the adapting and test stimuli.

To conclude, our results show that the effect of adaptation on the perceived numerosity of sequential stimuli generalizes across several different cross-modal combinations: the adaptation effect works irrespective of which modality is used to convey adaptor and test stimuli. Our findings thus expand previous results concerning the cross-modal effects of adaptation in numerosity perception and provide novel evidence for the existence of a truly amodal, generalized mechanism for the processing of numerosity.

## Data Availability Statement

All the datasets generated during the experiments described in this manuscript have been uploaded to Open Science Framework (OSF), and are accessible at the following link: https://osf.io/a47js/.

## Ethics Statement

The protocol of the present study (involving human participants) was reviewed and approved by the Comitato Etico Pediatrico Regionale—Azienda Ospedaliero-Universitaria Meyer—Firenze FI. The participants provided their written informed consent to participate in this study.

## Author Contributions

IT and RA designed the research, revised and edited the draft, and agreed on the final version of the manuscript. IT programmed the task, piloted the study, performed the research, analyzed the data in interaction with RA, designed the figures in interaction with RA, and wrote the first draft of the article. All authors contributed to the article and approved the submitted version.

## Conflict of Interest

The authors declare that the research was conducted in the absence of any commercial or financial relationships that could be construed as a potential conflict of interest.

## Publisher’s Note

All claims expressed in this article are solely those of the authors and do not necessarily represent those of their affiliated organizations, or those of the publisher, the editors and the reviewers. Any product that may be evaluated in this article, or claim that may be made by its manufacturer, is not guaranteed or endorsed by the publisher.
