# Supplementary material for: Evidence for an A-Modal Number Sense: Numerosity Adaptation Generalizes Across Visual, Auditory, and Tactile Stimuli
Source: Front Hum Neurosci. 2021 Aug 11;15:713565. doi: 10.3389/fnhum.2021.713565 (PMC8385665; doi:10.3389/fnhum.2021.713565)
Supplement: Supplementary file 1 [file Data_Sheet_1.docx]

Supplementary Material


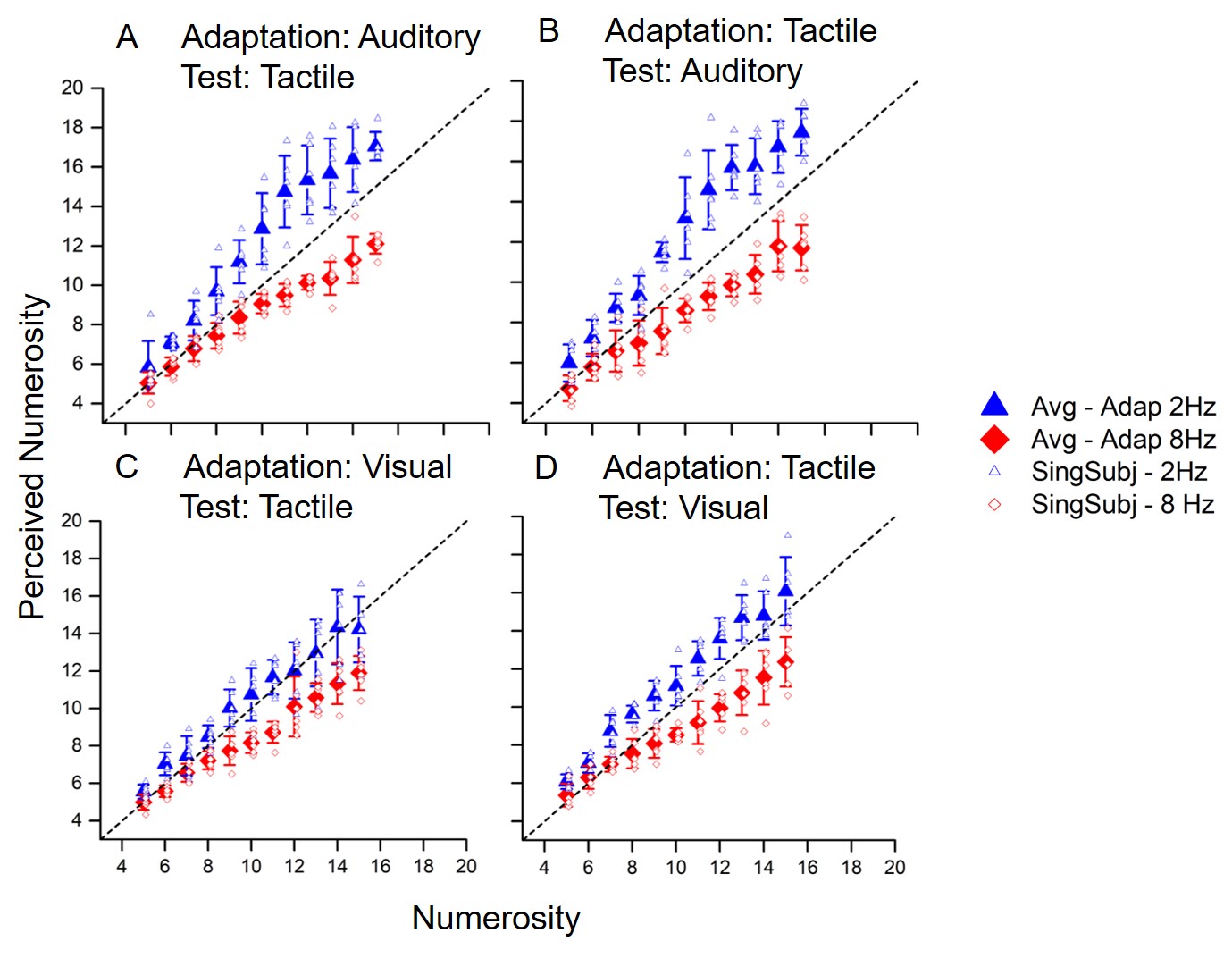


**Supplementary Figure 1. Perceived numerosity in different adaptation conditions, across the numerosity range.** (A) Perceived numerosity in the Auditory-Tactile condition, relative to the 2 Hz (blue) and 8 Hz (red) adaptation condition, plotted as a function of the physical numerosity of the stimuli (x axis). The filled symbols represent the average numerical estimates at each numerosity and in each adaptation conditions across the group. The small empty symbols represent individual data. (B) Average perceived numerosity in the Tactile-Auditory condition. (C) Average perceived numerosity in the Visual-Tactile condition. (D) Average perceived numerosity in the Tactile-Visual condition. Error bars are SEM.

**
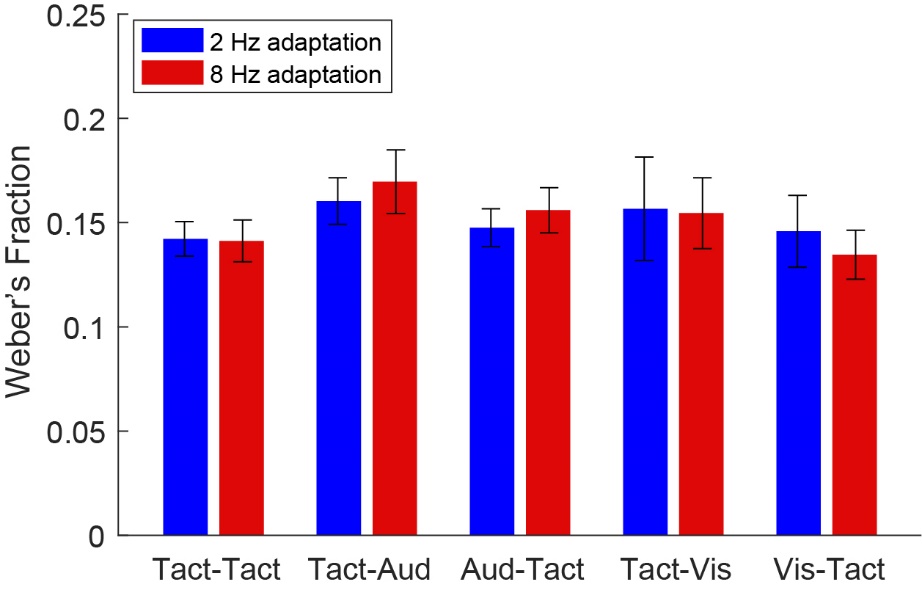
**

**Supplementary Figure 2. Average Weber’s fraction across the different adaptation conditions.** The average Weber’s fraction (WF) in the different conditions is shown individually for the 2 Hz adaptation condition (blue) and the 8 Hz adaptation condition (8 Hz). Error bars are SEM.
